# Prior Lung Inflammation Impacts on Body Distribution of Gold Nanoparticles

**DOI:** 10.1155/2013/923475

**Published:** 2013-01-13

**Authors:** Salik Hussain, Jeroen A. J. Vanoirbeek, Steven Haenen, Vincent Haufroid, Sonja Boland, Francelyne Marano, Benoit Nemery, Peter H. M. Hoet

**Affiliations:** ^1^Laboratory of Molecular and Cellular Responses to Xenobiotics, CNRS EAC 7059, Unit of Functional and Adaptive Biology (BFA), Sorbonne Paris Cité, University of Paris Diderot, 75 013 Paris, France; ^2^Lung Toxicology Research Unit, KU Leuven, 3000 Leuven, Belgium; ^3^Clinical Research Unit, National Institute of Environmental Health Sciences (NIEHS), NIH, Research Triangle Park, North Carolina NC 27709, USA; ^4^Louvain Centre for Toxicology and Applied Pharmacology, Catholic University of Louvain, 1200 Brussels, Belgium

## Abstract

*Introduction*. Gold- (Au-) based nanomaterials have shown promising potential in nanomedicine. The individual health status is an important determinant of the response to injury/exposure. It is, therefore, critical to evaluate exposure to Au-nanomaterials with varied preexisting health status. *Objective*. The goal of this research was to determine the extent of extrapulmonary translocation from healthy and inflamed lungs after pulmonary exposure to AuNPs. Male BALB/c mice received a single dose of 0.8 mg · kg^−1^ AuNPs (40 nm) by oropharyngeal aspiration 24 hours after priming with LPS (0.4 mg · kg^−1^) through the same route. Metal contents were analyzed in different organs by inductively coupled plasma-mass spectrometry (ICP-MS). *Results*. Oropharyngeal aspiration resulted in high metal concentrations in lungs (*P* < 0.001); however, these were much lower after pretreatment with LPS (*P* < 0.05). Significantly higher concentrations of Au were detected in heart and thymus of healthy animals, whereas higher concentrations of Au NPs were observed in spleen in LPS-primed animals. *Conclusions*. The distribution of AuNPs from lungs to secondary target organs depends upon the health status, indicating that targeting of distinct secondary organs in nanomedicine needs to be considered carefully under health and inflammatory conditions.

## 1. Introduction

Nanotechnologies have shown promising potentials in multiple sectors of everyday life. These advances span from material science to consumer products. More recently, various nanomaterials have proved themselves as excellent candidates for nanomedical applications. These range from diagnostics to drug and gene delivery applications. Gold (Au) is one of the major nanomaterials engineered for utilizations in medicine and electronics. The carrier properties of Au NPs make them promising candidates for delivering biological molecules into the cells thus making them an ideal platform for drug and gene delivery [1–4]. Au-based therapeutic strategies (hyperthermal therapy) mainly imply their role as heat-mediating objects (due to their strong light absorbing properties) to destroy particle-loaded cells/tissues [5, 6]. The absorbed light energy is dissipated into the particle surroundings leading to elevated temperatures in their vicinity. This hyperthermal property can further be used as therapeutic strategy to open drug carriers (polymer microcapsules) [7]. Moreover, Au NPs have shown promises as labelling/contrast agents (transmission electron microscopy/X-rays) and sensing agents. Another advantage of these materials is the possibility of utilizing previously mentioned properties at the same time (hyperthermal and photoacoustic imaging) to have combined schemes for the better evaluation of the biological phenomenon. Keeping in view the broad spectrum of *in vivo* applications of Au NPs, the potential deleterious effects of these NPs might become an issue which needs to be evaluated with care. Presently, there is a need to evaluate the potentials deleterious effects of Au NPs in both *in vitro* and *in vivo* conditions as a discrepancy exists in the literature about the cytotoxic effects of Au NPs on different types of cells [8, 9].

It has been shown that some NPs can cross physiological barriers, reach to secondary target organs, and may lead to unexpected outcomes [10, 11]. There exists a discrepancy in the existing literature about the extrapulmonary translocation of NPs/ultrafine particles [10–12]; however, the NP size dependence of translocation is an accepted fact [13, 14]. Preexisting respiratory disorders (e.g., inflammation) may modify the effects of NPs on the respiratory tract and can influence the amount of translocated material. Under normal conditions, lungs are often primed with endotoxins from the inhaled air [15]. We hypothesized that preexisting inflammation may influence the ability of Au NPs to pass through the pulmonary barrier and other organs in the body. To verify this hypothesis, we tested Au (40 nm) NPs in LPS-treated mice as an airway inflammation model.

## 2. Materials and Methods

### 2.1. Reagents

Tetrachloroauric acid (99.999%, 3H_2_O) was purchased from Aldrich. Citrate Tribasic dihydrate (95%, C_6_H_5_O_7_Na_3_, 2H_2_O) and LPS (*Escherichia coli* O55: B5) were obtained from (Sigma-Aldrich, Steinheim, Germany). The glassware and magnetic beads were always cleaned prior to use with freshly prepared aqua regia (1 : 3 HNO_3_ : HCl) followed by rinsing with ultrapure water (Millipore, conductivity: 0.8 mS·cm).

### 2.2. Animals

Male BALB/c mice (approximately 25 g, 6 weeks old) were obtained from Harlan (The Netherlands). The mice were housed in a conventional animal house with 12 h dark/light cycles. They received lightly acidified water and pelleted food (Trouw Nutrition, Gent, Belgium) ad libitum. All experimental procedures were approved by the Local Ethical Committee for Animal Experiments (Katholieke Universiteit Leuven, Leuvin, Belgium).

### 2.3. Au Nanoparticle Synthesis

Au NPs of 40 nm primary particle size were prepared in the laboratory (Institut d'Electronique Fondamentale UMR CNRS 8622, Université Paris-Sud, Orsay, France) by Turkevich method. Briefly, an aqueous solution of gold tetrachloroauric acid, with a weight content of 82.8 mg of gold, was heated until boiling point under vigorous stirring. Then, an aliquot of a 1% sodium citrate aqueous solution is added. Gold nanoparticles with average sizes of 39.8 nm were prepared by adjusting the ratio [AuCl_−4_]/[Citrate] from 0.4 to 1.3. After the introduction of the citrate solution, a purple colour appeared which then turned to ruby red. The solution was then stirred and kept at boiling conditions for another 45 minutes to complete the reduction process. In these experiments, NP suspensions (0.4 mg/mL, i.e., 0.8 mg/kg) stabilized in 2.5 mM sodium citrate tribasic dihydrate (vehicle) were utilized to treat the mice.

### 2.4. Au NP Characterization

These particles were thoroughly characterized for their physicochemical characteristics including morphology, zeta potential (*ζ*), size distribution, and hydrodynamic diameters.

#### 2.4.1. Transmission Electron Microscopy (TEM)

Microscope measurements were performed using a Philips CM30 TEM (Philips FEI, Eindhoven, The Netherlands) operating at 300 kV. Small volumes of sample were deposited on copper mesh grids and covered with carbon coating films. The samples were then dried under an N_2_ atmosphere in a glove box.

#### 2.4.2. Dynamic Light Scattering

Dynamic light scattering (DLS) measurements were performed with a Brookhaven 90 Plus Nanoparticle Size Distribution Analyzer (scattering angle 90°, wavelength 659 nm, power 15 mW; Brookhaven Instruments Ltd, Redditch, UK). Correlation functions were analyzed using the Clementine package (maximum entropy method) for Igor Pro 6.02A (WaveMetrics, Portland, OR, USA). This resulted in intensity-weighted distribution functions versus decay times. By converting the decay times with instrument parameters and physical parameters to hydrodynamic diameters, an intensity-weighted size distribution is obtained. A log-normal fit was applied to each population, resulting in the intensity-weighted average hydrodynamic diameter of the population. Mass- and number-weighted distributions were estimated using the Rayleigh scattering approximation and a correction factor for the form factor of spherical particles.

#### 2.4.3. *ζ* Potential Measurements

*ζ* potential measurements were performed on the same NP solutions as used for DLS. Au and *ζ* potential were measured with a Brookhaven 90Plus/ZetaPlus instrument applying electrophoretic light scattering. A primary and reference beam (659 nm, 35 mW) modulated optics and a dip-in electrode system were used. The frequency shift of scattered light (relative to the reference beam) from a charged particle moving in an electric field is related to the electrophoretic mobility of the particle. The Smoluchowski limit was used to calculate the *ζ* potential from the electrophoretic mobility.

### 2.5. LPS Treatment

LPS (*Escherichia coli* O55: B5, Sigma Aldrich) was suspended in HBSS and administered 10 *μ*g/mouse (0.4 mg/kg). The dose of LPS was based on information available in the literature; the amount given induces only local (pulmonary) inflammation [16].

### 2.6. Experimental Design

**Figure 1:**
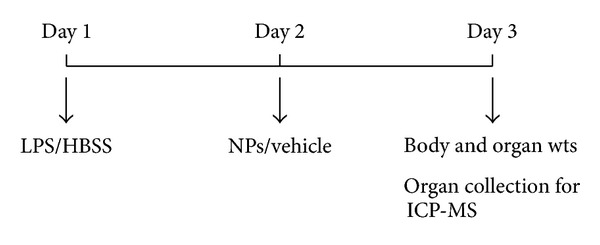
Experimental design. Animals (*n* = 4) were given 10 *μ*L of LPS or its vehicle (HBSS) on day 1 by oropharyngeal route. On day 2, animals were administered 40 *μ*L of NP suspension or vehicle for NPs (2.5 mM trisodium citrate) by the same route. Twenty four hours later, animals were weighed and sacrificed, organs were collected, wet organ weights were measured, and samples were processed for ICP-MS analysis.

On day, 1 animals were exposed to 10 *μ*g (10 *μ*L) of LPS or HBSS (vehicle for LPS) by oropharyngeal aspiration (Figure 1). On day 2, animals were administered 40 *μ*L of 0.4 mg/mL (0.8 mg/kg) NP suspensions or vehicle through same route. On day 3 (24 hours later), the animals were sacrificed, blood was collected through retro-orbital plexus, and the organs were removed. Organs (brain, thymus, lung, heart, liver, spleen, kidney, and testis) were weighed to obtain wet weight. Each experimental group comprised of 4-5 animals.

### 2.7. Metal Content Analysis

After weighing, the organs were placed in glass tubes and digested using 2 mL pure 60% nitric acid (Sigma-Aldrich). The tubes were placed in a water bath at 80°C until all the tissues were solubilized. The samples were then analyzed for metal contents. The analytical determination of Au in samples was performed by inductively coupled plasma-mass spectrometry (ICP-MS) using Agilent 7500cx. Samples were diluted 100 times with a basic diluents (butanol 2%, EDTA 0.05%, NH_4_OH 1%, and triton 0.05%) containing internal standards. The quantification of Au was performed using the unique 197Au isotope and 193Ir as an internal standard in the “nogas mode” (standard mode).

### 2.8. Statistical Analysis

Data are presented as mean ± SD where *n* = 3-4 animals per group. All groups were tested for normality using the Kolmogorov-Smirnov normality test. Since our data were normally distributed, we applied an analysis of variance (ANOVA) followed by Tukey's test for multiple comparisons using Graphpad (Graphpad Prism 4.01, Graphpad Software Inc., San Diego, USA). A level of *P* < 0.05 (two tailed) was considered significant.

## 3. Results

### 3.1. Au NP Characteristics

**Figure 2:**
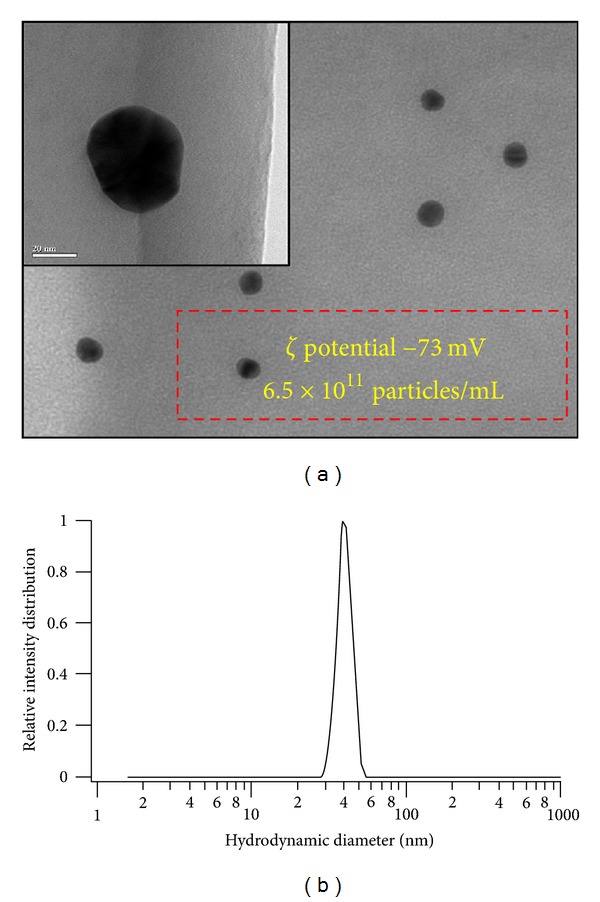
Characterization of Au NPs after suspension in 2.5 mm sodium citrate. (a) Transmission electron microscopic (TEM) image of 40 nm Au NPs. Inset shows a single particle at higher magnification (bar 20 nm). Box indicating *ζ* potential of these Au NP suspensions (in 2.5 mM sodium citrate) and particle number per mL. (b) Dynamic Light Scattering (DLS) analysis of Au nanoparticle suspension showing single population of Au nanoparticles, having 40 nm hydrodynamic diameter.

Au NP suspension optical spectroscopy analysis of Au NP revealed a single Plasmon peak around 520 nm. TEM analysis revealed spherical morphology of Au NPs (Figure 2(a)), and size distribution analysis indicated a single peak of Au NPs with 40 nm hydrodynamic diameter (Figure 2(b)). The *ζ* potential measurements in 2.5 mM sodium citrate solution (vehicle for animal exposure) indicated that Au NPs had −73 mV showing that electrostatic repulsions are important factor in stabilising the suspensions. This further confirmed the stabilising effect of citrate solution as these NPs showed lower negative *ζ* potentials in water (data were not shown).

### 3.2. Animal Study

**Figure 3:**
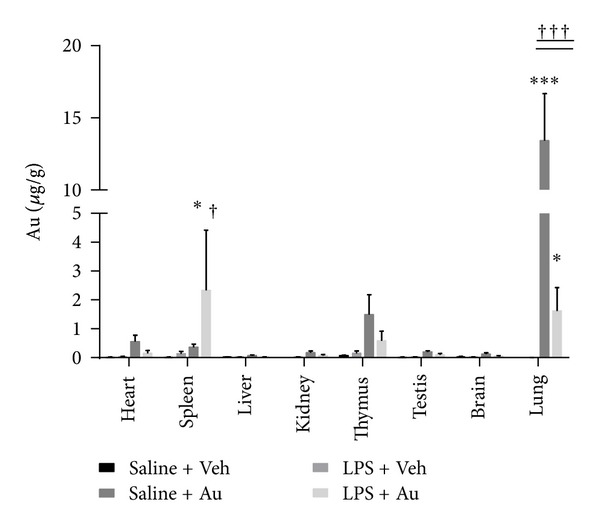
Body distribution of Au NPs with or without pretreatment with LPS. The concentration of Au NPs (*μ*g·g^−1^ relative organ weight) in lungs (a) in different secondary organs (b) of animals with or without pretreatment with LPS. ∗ represents statistically different from respective (saline/LPS) control group without NPs and † represents statistically different from NP-treated group without LPS, *P* < 0.05 (two tailed). ***P < 0.01 and ^†††^ *P* < 0.01.

Only lung relative weights differed significantly between saline and LPS-treated groups (data were not shown) due to inflammation and oedema caused by LPS in the lungs. Significant increases in metal concentration (relative to background values in an untreated group) in the lungs of the animals were detected by ICP-MS (Figure 3). After priming with LPS, lower amounts were detected in the lungs of primed animals as compared to those of nonprimed animals (Figure 3). In nonprimed animals, higher amounts of Au were detected in heart and thymus. However, a significant increase after LPS priming was observed in spleen (Figure 3 inset).

## 4. Discussion

This study was designed to assess the extrapulmonary translocation of Au NPs in a pulmonary inflammation model. Quantitative data indicated that 81% ± 10% of the aspirated dose remains in the lungs of the healthy animals, with 3% ± 3% and 6 % ±  2% being found in heart and thymus, respectively. However, in LPS primed animals, only 25% ± 8% was detected in lungs, with 7% ± 5% detected in spleen and 3% ± 1% detected in thymus. Interestingly, we found that in LPS-primed animals, the amount of Au which reach spleen is 5-6 fold higher than found in healthy animals.

**Figure 4:**
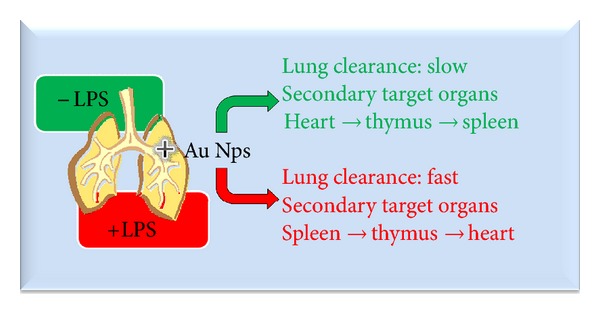
Schematic overview of the results presented in this study.

We demonstrate here that even in healthy animals, Au NPs reach secondary target organs after lung exposures. Moreover, striking differences in the target organs (spleen versus heart) between LPS exposed and unexposed animals is of particular interest. A graphical overview of the findings is presented in Figure 4. Recently, several reports have emphasized the potential applications of Au NPs in nanomedicine, but the possibilities of such secondary effects have not been illustrated. The mechanisms of translocation through the air-blood barrier remains unclear: epithelial uptake may occur and cause damage to epithelial cells; electron microscopic study demonstrated UFPs passage via the clefts between the alveolar epithelial cells in healthy conditions. As to the mechanisms of damages of the alveolar wall by LPS, it is suggested that macrophages and neutrophils activated by LPS release free radicals resulting in the degeneration of the air-blood barrier. Translocation of NPs from the air-blood barrier to the capillary lumen may take place through the degenerated structures with acute inflammatory condition [15, 17].

Is has also been observed that NPs potentiate an inflammatory response in subjects with lung inflammation. In view of the impact on (innate and adaptive) immunity, NPs influence cell populations, such as macrophages/monocytes, neutrophils, dendritic cells, natural killer cells, and lymphocyte [18–20]. The preexposure stimulation of the immune system with LPS, resulting in significant changes in the spleen, probably lays on the basis of the different distribution in inflammatory conditions [21].

An important fact is that these data are obtained using realistic NP doses (doses which do not induce inflammation in the lungs of healthy animals) contrary to the literature reports which used huge amounts of NPs to observe systemic translocation. Previously, it was demonstrated that the majority of the administered Au NPs are retained within the healthy lung and only small portion reaches systemic circulation after inhalation exposure [22].

We have recently reported that in the experiments done in parallel with the same dose of Au NPs given through same route did not induced inflammation (neither an increase in bronchoalveolar lavage fluid cellularity nor cytokines) [23]. The reason for the increase in Au in the spleen after LPS exposure is not clear. LPS exposure provokes an inflammatory response, leading to influx of inflammatory cells in the lungs resulting in an increase in phagocytosis of deposited material by macrophages. These macrophages might play a central role in the transport and clearance of the Au NPs, thus explaining the lower amounts material after LPS exposure [24]. Moreover, we have recently showed increased numbers of Au laden macrophages in broncho-alveolar lavage fluids of “asthmatic” animals in a mouse model of diisocyanate-induced asthma [23]. It has been demonstrated that macrophages (in particular Kupffer cells) play the most important role in the clearance of intravenously injected Au NPs [25]. We are currently evaluating these mechanisms in detail, but we consider it timely and important to share our preliminary observations with the scientific community. However, in another study, it was shown that 15-day inhalation chamber exposure to Au NPs results in accumulation of significant amounts of Au in spleen along with many parts of digestive and cardiovascular system in rats [26]. Recent studies demonstrated the presence of gold nanoparticles and nanorods in spleen after intravenous exposures [27]. The differential interaction with lung lining fluid (in case of inhalation exposure) and blood proteins (in case of intravenous exposure) were postulated to be the reasons for differential body distributions of Au NPs in inhalation versus intravenous exposures [27]. We show here that Au NPs can reach spleen even in case of lung exposure. It could be speculated that there might exist the possibilities of immunomodulatory/immunotoxic effects of Au NPs. Moreover, it is noteworthy that it has been already shown that Au reduces the antigen presentation and autoimmune reactions in rheumatoid arthritis [28]. However, in depth, mechanistic studies are needed to elucidate whether Au NPs exposure that can also result in similar outcome is unknown.

The shortcomings of the present study did not include directly measuring inflammatory response in the lungs (relying on the literature data/previous experience with the LPS dose to induce inflammation) and housing animals in metabolic cages to comprehensively estimate the clearance of NPs from the body and to account for the dose lost from the LPS-primed lungs. Moreover, the role of NP physicochemical characteristics, surface modification/functionalization, and protein corona need in-depth evaluations. Recently, It has been shown that protein corona can significantly modify the responses to biomedical nanoparticles and its complementary factor cell vision should also be considered [29].

## 5. Conclusion and Perspectives

In conclusion, our results confirm the hypothesis of particle translocation from the lungs to secondary organs like spleen, heart, and thymus. The observation that NPs target different organs depending on the health status of the animal warrants further studies to understand the mechanisms involved in this process and possible consequences. There is an urgent need of both *in vivo* and *in vitro* mechanistic studies to better understand the possible differences in interactions of Au NPs with different organ systems in the body. A particular focus must be made to understand the unexpected outcomes of Au NP exposures in animal models of different diseases. Moreover, mechanistic studies are warranted to understand the interaction of Au NPs with immune system.

## 6. Executive Summary

- Au NPs can cross the air-blood barrier in both healthy and inflamed lungs.
- Preexisting inflammation alters the body distribution pattern of Au NP.
- Distinct organ targeting in case of inflamed lungs indicates the need to evaluate the consequences of NP administration in diseases status.
